# mHealth-Assisted Detection of Precursors to Relapse in Schizophrenia

**DOI:** 10.3389/fpsyt.2021.642200

**Published:** 2021-05-31

**Authors:** Benjamin Buck, Kevin A. Hallgren, Andrew T. Campbell, Tanzeem Choudhury, John M. Kane, Dror Ben-Zeev

**Affiliations:** ^1^Department of Psychiatry and Behavioral Sciences, Behavioral Research in Technology and Engineering (BRiTE) Center, University of Washington, Seattle, WA, United States; ^2^Department of Computer Science, Dartmouth College, Hanover, NH, United States; ^3^Department of Information Science, Cornell University, Ithaca, NY, United States; ^4^The Zucker Hillside Hospital, Northwell Health, Glen Oaks, NY, United States; ^5^Donald and Barbara Zucker School of Medicine at Hofstra/Northwell, Hempstead, NY, United States

**Keywords:** technology, mobile health, schizophrenia, relapse, ecological momentary assessment

## Abstract

Theoretical views and a growing body of empirical evidence suggest that psychiatric relapses in schizophrenia-spectrum disorders (SSDs) have measurable warning signs. However, because they are time- and resource-intensive, existing assessment approaches are not well-suited to detect these warning signs in a timely, scalable fashion. Mobile technologies deploying frequent measurements—i.e., ecological momentary assessment—could be leveraged to detect increases in symptoms that may precede relapses. The present study examined EMA measurements with growth curve models in the 100 days preceding and following 27 relapses (among *n* = 20 individuals with SSDs) to identify (1) what symptoms changed in the periods gradually preceding, following, and right as relapses occur, (2) how large were these changes, and (3) on what time scale did they occur. Results demonstrated that, on average, participants reported elevations in negative mood (*d* = 0.34), anxiety (*d* =0.49), persecutory ideation (*d* =0.35), and hallucinations (*d* =0.34) on relapse days relative to their average during the study. These increases emerged gradually on average from significant and steady increases (*d* = 0.05 per week) in persecutory ideation and hallucinations over the 100-day period preceding relapse. This suggests that brief (i.e., 1–2 item) assessments of psychotic symptoms may detect meaningful signals that precede psychiatric relapses long before they occur. These assessments could increase opportunities for relapse prevention as remote measurement-based care management platforms develop.

## Introduction

Relapses in schizophrenia-spectrum disorders (SSDs) are devastating. In addition to disrupting the lives of individuals experiencing them, each relapse event cumulatively increases the likelihood of subsequent relapses (1), dysfunction (2), poor treatment response (3, 4), and suicide (5). Relapses in SSDs often culminate in psychiatric hospitalizations, which are among the costliest elements in healthcare (6). This is a key reason why SSDs are a leading cause of global disability and healthcare costs (7), costing more than $102 billion worldwide (8–10). Relapses are typically preceded by smaller elevations in symptoms (e.g., delusions, hallucinations, suspiciousness, anxiety) that are potentially detectable before full-blown relapse (11, 12). Intervening to address these symptoms rapidly has the potential to help mitigate the likelihood of relapse and associated sequelae.

Existing approaches used to detect relapse risk are limited by problems with feasibility. Symptoms are typically evaluated through clinical impressions, in-person interviews, or clinician-administered rating scales, which require direct contact with a trained provider. Depending on in-person interactions limits potential reach and scalability, as structured symptom assessments are time and resource intensive, and because the majority of individuals with serious mental illnesses are not regularly engaged in care (13). Further, rater-administered scales require respondents to summarize their experiences over long periods of time. This approach is susceptible to inaccuracies related to memory errors/recall bias (14), interpretive errors (15), or assessment demand characteristics (16).

Mobile devices can be used to deploy a series of brief, self-report measures administered during patients' day-to-day lives [i.e., ecological momentary assessment or EMA; (17)] using mobile technologies that many people with SSDs already have (18). These approaches not only have the potential to improve detection of relapse risk but also to characterize changes that occur in the post-relapse period as well, and to establish trajectory models. Assessment systems using EMA [(19) for a review] are feasible and acceptable to patients with chronic SSDs (20–22) as well as early psychosis (23, 24). The information provided by EMA could be useful in clinical settings aiming to detect symptom increases that signal impending relapse or to track recovery during high-risk periods (i.e., after hospitalizations). New approaches using weekly assessments of aggregate warning signs collected via mobile SMS (25, 26) have demonstrated that rather than occurring in a 2–4 week period as has been previously hypothesized, patients report gradual increases that begin 2 months before a relapse (27). Few studies have assessed changes in individual symptoms that precede and follow relapses with mobile devices. A more granular understanding of the symptom increases that precede relapses could help identify which individual symptoms change prior to relapse, what degree of increases might indicate impending relapse, and on what time scale these changes might be detected. This information could inform the further development of assessments aiming to better detect and prevent the onset of relapse and to inform attempts to understand relevant underlying biological/pharmacological processes.

Ben-Zeev and colleagues deployed a multi-modal mobile assessment system—CrossCheck—in a sample of individuals with schizophrenia for 12 months (28–30). This system administered EMA scales up to three times per week over this period. The present study aims to determine whether a brief report of individual symptoms assessed via EMA detects changes occurring before, during, and after psychiatric relapses. We operationalize this approach by examining (1) whether and to what degree EMA responses assessing symptoms change before relapse, (2) whether mean values predicted by those models on the day of relapse differ from participant averages throughout the study period, and (3) whether and to what degree responses change following relapse.

## Materials and Methods

### Participants

Participants were recruited from a large psychiatric hospital in New York via on-site flyers as well as study staff review of hospital electronic medical records. The clinicians of potentially eligible individuals were contacted by a member of the research team and were asked to provide these individuals with a study description. A member of the study team reached out to participants who gave their clinician authorization to pass along their names and contact information. Prospective participants received a detailed description of the study before being evaluated for eligibility. Inclusion criteria were (1) being 18 years or older; (2) having a diagnosis of schizophrenia, schizoaffective disorder, or psychosis not otherwise specified; and (3) the occurrence of an inpatient psychiatric hospitalization, daytime psychiatric hospitalization, outpatient crisis management visit, or short-term psychiatric hospital emergency room visit within the last 12 months. Individuals were excluded if they (1) had hearing, vision, or motor impairments that would interfere with the use of a smartphone (determined using a demonstration smartphone during screening); (2) had a reading level below 6th grade [determined using the reading section of the Wide Range Achievement Test; (31)]; or (3) a lack of competency to consent.

### Procedure

Participants were randomized to one of two conditions: (1) the intervention condition (i.e., access to the CrossCheck system and as needed follow-up for 12 months), or (2) treatment as usual (i.e., no change to participants usual care over the same period). Participants in both conditions were asked to attend in-person data collection visits every 3 months. At these visits, participants were administered the Brief Psychiatric Rating Scale [BPRS; (32)], a 24-item interview-rated assessment of an array of symptoms of schizophrenia-spectrum disorders. Baseline characteristics of participants—including those who experienced a relapse and those who did not during the study period (33)—are reported elsewhere.

Data for the present report are drawn from participants (*n* = 61) randomized to the CrossCheck condition. All participants in this condition were asked to carry a study device with them for 12 months. These devices were Samsung Galaxy S5 Android smartphones with unlimited data plans. Participants were instructed to charge the device each night while sleeping to reduce instances of missing data. This device had an integrated mobile monitoring assessment system called CrossCheck pre-installed. CrossCheck deployed EMA self-report scales and collected data from sensors that are embedded in most contemporary smartphones including accelerometers and Global Position System (GPS). A full description of the study software (28–30) as well as previous results involving passive sensing are reported elsewhere (33, 34).

### Ecological Momentary Assessment (EMA)

CrossCheck prompted participants to complete a 10-item EMA self-report survey three times per week (i.e., all Mondays, Wednesdays, and Fridays), with the prompt, “Just checking in to see how you've been doing over the last few days.” A full list of EMA items can be found in Table 1; response options to each question ranged from 0 (*not at all*) to 3 (*extremely*). In addition to symptoms of psychosis, EMA items also assessed overall mental health (e.g., stress, depression, hopefulness, calmness, clarity of thought), and functioning (e.g., socialization, sleep). For the present study, we identified five variables based on research (11, 12, 27) suggesting that negative mood, anxiety, hallucinations, and delusions are elevated in assessments that precede relapses. Two of those items—persecutory ideation, and self-reported sleep quality—were assessed with a single item, while three—negative mood (average of the “depression” item and reverse-coded “hopefulness” item), anxiety (“stress” item and reverse-coded “calm” item), and hallucinations (average of “seeing things” and “voices” items)—were assessed as composite scores. These items are listed in Table 1.

**Table 1:** Overview of EMA items gathered by CrossCheck examined in the present study.

**Prompt: Just checking in to see how you've been doing over the last few days**.
**Negative affect**
Have you been DEPRESSED?
Have you been HOPEFUL about the future? (R)
**Anxiety**
Have you been feeling STRESSED?
Have you been feeling CALM? (R)
**Hallucinations**
Have you been bothered by VOICES?
Have you been SEEING THINGS other people can't see?
**Persecutory ideation**
Have you been worried about people trying to HARM you?
**Sleep**
Have you been SLEEPING well?

*0, Not at all; 1, A little; 2, Moderately; 3, Extremely. R, Reverse scored in the present analysis*.

### Assessment of Relapse

Hospital electronic health record data were made available to the research team for tracking psychiatric relapses during the study period. Trained study staff identified potential relapses from this record with final confirmation by the study PI (DBZ). The following events, reported during study assessments or recorded in the medical record, were designated as relapses: psychiatric hospitalization, significant increase in the level of psychiatric care (i.e., frequency and intensity of services, dosage increase or additional medicines prescribed) together with either an increase of 25% from baseline on BPRS total score, suicidal or homicidal ideation that was clinically significant in the investigators' judgment, deliberate self-injury, or violent behavior resulting in damage to another person or property (35). When documentation of the relapse date was unavailable (e.g., instances of self-reported suicidal ideation that did not lead to hospitalization or suicide attempt) assessors gathered data about the specific relapse event date directly from study participants.

### Data Analytic Plan

We deployed latent growth curve analysis within a multilevel modeling framework to model gradual changes in symptoms over 100-day periods before and after relapse, as well as sudden changes in symptoms before vs. after relapse. Specifically, we modeled discontinuous growth curves to characterize separate trajectories of changes in symptoms over time before and after relapse and sudden changes in symptoms on the day of relapse. Similar analytic approaches have been used to model gradual and sudden changes in symptoms for other disorders (e.g., substance use disorders) with respect to other significant events [e.g., initiating abstinence from alcohol (36) or tobacco (37)]. Growth curve models included fixed effects for (a) linear change over time in the 100 days before relapses, (b) linear change for the 100 days following the relapse, and (c) sudden changes in expected values from the end of the pre-relapse period to the beginning of the post-relapse period. Random subject-level terms for all three of these effects were also entered into this model, as well as a random intercept, to account for between-subject heterogeneity in symptom trajectories. Growth curve models were analyzed within a multilevel modeling framework (repeated measures nested within persons) and a separate model was fit for each symptom measure. Models were fit using maximum likelihood estimation, which reduces bias and improves precision when data are missing at random or missing completely at random (38).

Time variables for modeling gradual change were scaled such that a one-unit increase in time reflected a 1-week period for the pre or post relapse period, and the time variable reflecting sudden change was dummy coded as 0 during the pre-relapse period and 1 in the post-relapse period. Thus, model coefficients indicated the rate of change in symptoms per week during the pre or post relapse period, and the amount of sudden change in symptoms when transitioning from pre relapse to post relapse (beyond what was attributable to the gradual change over time trajectories). Because our analyses aimed to examine gradual changes over periods consistent with previous research (27), our models included EMA data within 100 days before or after relapse.

For individuals with more than one psychiatric relapse (*n* = 4), each relapse was included in the analysis and time variables reflecting “weeks to relapse” and “weeks from relapse” were split at the halfway point between the two relapses. In a sensitivity analysis, we conducted analyses a second time with second relapses excluded for these four participants and an identical pattern of results were found. EMA variables were person-mean centered such that the zero point in all models represented the participant's average EMA response during the full study period. This allowed for interpretation of intercepts as the model-inferred difference between participants' expected symptom ratings on the day of relapse compared to their overall average response to that EMA item during the study period.

## Results

### Relapses

Twenty-seven relapse events occurred during the study period across 20 participants; models predicting relapse examine this subset of individuals. Of those events, they were characterized (non-mutually exclusively) as follows: psychiatric hospitalization (*n* = 22, 81.48%), increased frequency of services (*n* = 7, 25.93%), increased medication and BPRS increase (*n* = 6, 22.22%), suicidal ideation (*n* = 4, 14.81%) homicidal ideation (*n* = 1, 3.70%), self-injury (*n* = 2, 7.41%), violence (*n* = 1, 3.70%). At baseline, participants who went on to relapse did not significantly differ from those who did not relapse with regard to age, gender, race, ethnicity, diagnosis, or number of past psychiatric hospitalizations (past year or lifetime).

**Figure 1:**
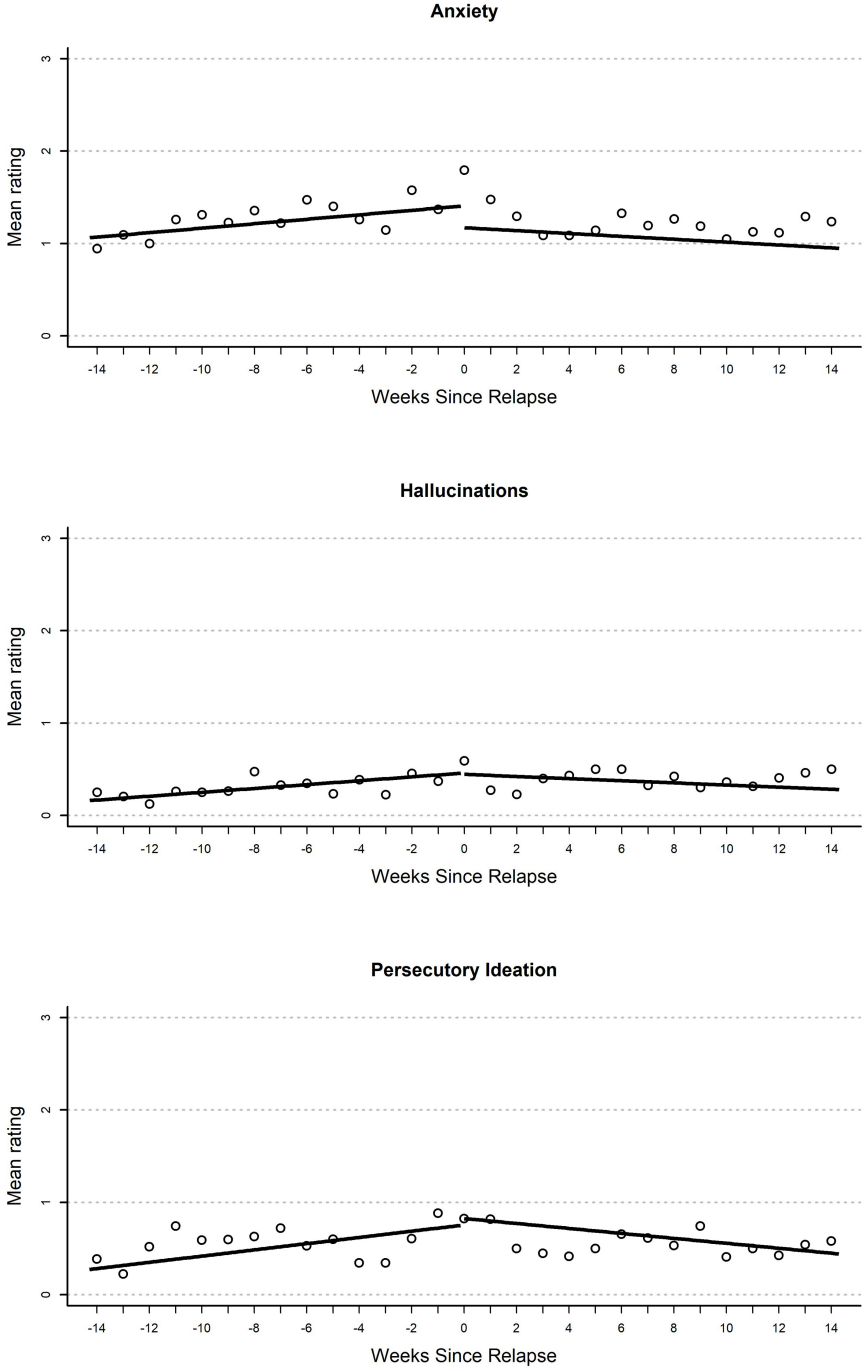
Plot of mean EMA rating for anxiety **(top)**, hallucinations **(middle)**, and persecutory ideation **(bottom)** variables by weeks until or since the relapse event.

**Figure 2:**
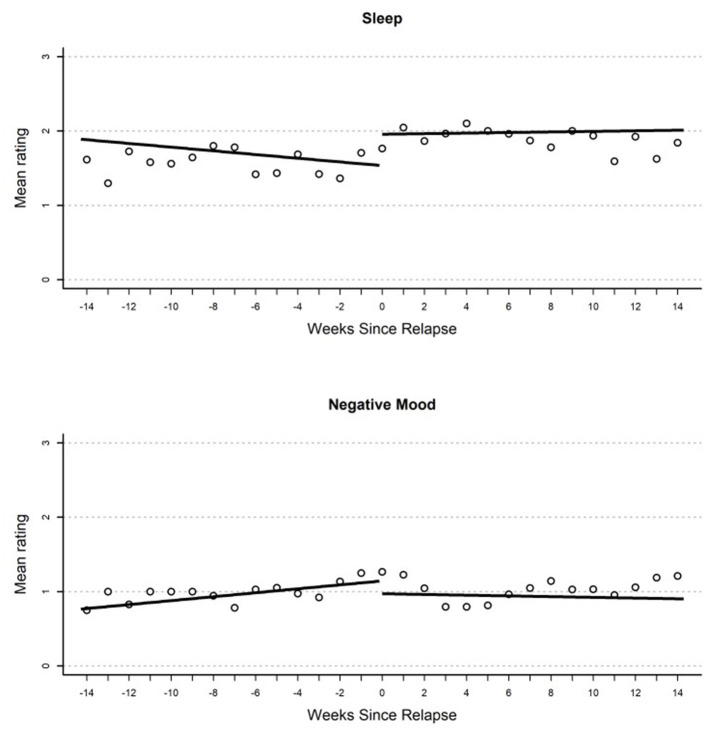
Plot of mean EMA rating for sleep **(top)** and negative mood **(bottom)** by weeks until or since the relapse event.

All model estimates are provided in Tables 2, 3. Parameter estimates in each model represent (1) the average participant-centered EMA value on the relapse day (“Intercept”), (2) immediate changes that occur from the end of the pre-relapse period to the beginning of the post-relapse period (“Immediate change”), (3) rates of change in the EMA values per week before relapse (“Gradual pre-relapse change”), and (4) rates of change in the EMA values after relapse (“Gradual post-relapse change”). All model-implied growth curves are displayed in Figures 1, 2.

**Table 2:** Full growth curve models for negative mood, anxiety, and sleep variables.

	**Negative mood**			**Anxiety**			**Sleep**		
	**B**	**SE**	***p***	**B**	**SE**	***p***	**B**	**SE**	***p***
Intercept	0.17	0.07	0.03^*^	0.23	0.07	0.002^**^	−0.28	0.16	0.093
Immediate change	−0.15	0.10	0.18	−0.21	0.11	0.056	0.43	0.22	0.07
Gradual pre-relapse change	0.02	0.01	0.054	0.02	0.01	0.08	−0.03	0.02	0.17
Gradual post-relapse change	−0.00	0.01	0.54	−0.02	0.01	0.11	0.00	0.01	0.75

* *p < 0.05*,

** *p <0.01*.

**Table 3:** Full growth curve models for persecutory ideation and hallucinations variables.

	**Persecutory ideation**			**Hallucinations**		
	**B**	**SE**	***p***	**B**	**SE**	***p***
Intercept	0.20	0.06	0.002^**^	0.15	0.05	0.01^*^
Immediate change	0.05	0.14	0.71	−0.04	0.07	0.61
Gradual pre-relapse change	0.03	0.01	0.002**	0.02	0.01	0.02*
Gradual post-relapse change	−0.02	0.01	0.08	−0.01	0.01	0.30

* *p < 0.05*,

** *p < 0.01*.

### Negative Mood

Results from growth curve models examining change in EMA report of negative mood over time are reported in the first heading of Table 2. On the day of relapse, participants had significantly more negative mood compared to their average in the study (*d* = 0.34). Overall, this suggests a significantly elevated negative mood when relapse occurred (see Figure 1). There were no other significant effects in the model; however, there was a non-significant (*p* = 0.054) gradual increase in negative mood (*d* = 0.04 per week) that over the 100-day pre-relapse period.

### Anxiety

Results from growth curves examining anxiety are reported in the second heading of Table 2. On the day of relapse, participants had model-implied ratings that indicated a statistically significant elevation in anxiety (*d* = 0.49). There were no other significant terms in the model. Overall, this provides support for elevations when relapse occurred (see Figure 1). There was a nominal gradual increase that occurred during the 100-day pre-relapse period (*d* = 0.04 per week, *p* = 0.075), as well as an immediate decrease when the relapse occurred (*d* = −0.45, *p* = 0.06), but both of these effects were non-significant.

### Sleep

Parameter estimates from growth curves related to sleep are reported in the third heading of Table 2. There were no significant effects in the model. There were two non-significant effects of similar magnitude including a reduction in reported quality of sleep on the day of relapse relative to average in the study period (*d* = 0.41), as well as an immediate increase in sleep quality (*d* = 0.64) when the relapse period began.

### Persecutory Ideation

Growth curve models examining persecutory ideation are reported in the first heading of Table 3. On the day of relapse, models inferred an elevated persecutory ideation score relative to participants' mean response (*d* = 0.35). This resulted from a significant linear increase during the 100-day pre-relapse period (*d* = 0.05 per week). Overall, this curve seems to suggest a steady but small increase in persecutory ideation over time before relapse and an elevation as the relapse occurred. There were no other significant effects in this model; however, there was a non-significant (*p* = 0.08) negative slope in the post-relapse period (*d* = 0.04).

### Hallucinations

Parameter estimates from growth curves related to hallucinations are reported in the second heading of Table 3. On the day of relapse, models inferred an elevated rating of hallucinations relative to the participant mean (*d* = 0.34). This resulted from a significant positive slope in the 100-day pre-relapse period (*d* = 0.05 per week). No significant immediate change was found from the pre-relapse to post-relapse period or during the post-relapse period. Overall, this model suggests a steady increase in hallucinations in pre-relapse period, as well as an elevation relative to average when relapse occurs (see Figure 2).

## Discussion

Results demonstrated that a brief mobile EMA system detected changes in self-reported mood and psychotic symptoms that occurred in the period preceding relapse, both gradually over time before the relapse and immediately as psychiatric relapses occur. A series of growth curve models suggested that there were elevations in negative mood (*d* = 0.34), anxiety (*d* = 0.49), persecutory ideation (*d* = 0.35), and hallucinations (*d* = 0.34) on relapse days relative to average, and steady increases in persecutory ideation and hallucinations were evident in the 100-day period preceding relapse. These increases represented a small effect (*d* = 0.05 for both hallucinations and persecutory ideation) each week for the duration of the pre-relapse period. This suggests that brief assessments (i.e., 1–2 items) may register changes in each of these domains that occur in the 100 days that precede psychiatric relapses. Brief assessments of persecutory ideation and hallucinations, administered multiple times per week, appear to detect a signal that could indicate risk.

In general, the present study provides preliminary support for the validity of EMA in providing information relevant to consequential clinical events. A brief EMA questionnaire appeared to detect changes in relation to meaningful and impactful clinical events. Because the financial and resource cost to administer EMA questionnaires is so low, the growing evidence supporting the utility of this information makes a compelling case for the use of remote monitoring in routine clinical care. The questionnaire deployed in the present study is brief (i.e., <2 min to complete), technologically simple, and does not require clinical staff to administer or calculate total scores. Indications of risk from EMA could trigger clinician outreach, indicate an increase in in-person sessions, or be used to encourage deployment of evidence-based mHealth self-management interventions directly to patients (39, 40). Our results suggested that low-level symptoms appeared to build up in the period leading up to relapse. Such subtle changes that may not be noticeable to individuals could potentially be detected with routine monitoring systems. Clinical interventions aiming to prevent or reduce the impact of psychiatric relapses may benefit from targeting individuals who sustain increases in these domains over time. Interventions that provide support and self-management strategies, even in response to small symptom increases, could provide benefits that prevent a costly and debilitating full relapse.

One limitation of the present study pertains to the size of the sample, as only 20 participants in the study relapsed during the study period, this limits statistical power to identify population-level trends in self-reported symptoms. While the lack of significance of these effects precludes further interpretation, there were several effects that fell just below (i.e., *p* <0.10) the threshold for statistical significance: gradual increases in the pre-relapse period in negative mood and anxiety, reductions in reported quality of sleep at the time of relapse, immediate post-relapse reductions in anxiety and sleep problems, and gradual post-relapse reductions in persecutory ideation. The study's lack of power may have reduced likelihood of finding significance. Future studies with larger samples, longer EMA periods, and more frequent assessments have the potential to clarify these areas. Second, as our EMA was administered on Monday, Wednesday, and Friday each week for the study period, there are many days during which participants did not provide any data. It is possible periods of missing data are aligned with precisely the periods during which participants are at greatest risk for relapse, as it may be difficult to complete EMA questionnaires when experiencing severe symptoms. Future longitudinal studies would be strengthened with a greater frequency of assessments, which could be feasible in light of the fact that participants with SMI in EMA studies report that they do not find these assessments to be burdensome (22, 24). While the questionnaire deployed assessed a range of symptoms, it was not designed for the purpose of assessment of early warning signs. Other systems examining EMAs have developed questionnaires expressly for this purpose (27). Importantly, during the CrossCheck study, members of the study team reached out to several participants who appeared at risk of relapse. Though clinically and ethically indicated in the study protocol, it is possible that taking these steps reduced the number of relapses during the study period and thus attenuated the strength of the relationships between EMA self-reported symptoms and relapse. Last, research examining emerging technologies using EMA along with passive sensors in this population has noted that the application of these data may be most useful ideographically and not only a population scale (41, 42). While the present study took such a population-based approach, this does not rule out idiosyncratic patterns (i.e., “relapse signatures”) (28) that characterize specific individualized risk. As clinical EMAs continue to expand into research and routine clinical care, future studies should continue to characterize trajectories of symptom change that precede and follow relapses in larger samples and with greater granularity.

The present study characterizes in greater depth the temporal changes in symptoms that characterize schizophrenia-spectrum disorders. In addition to reporting elevations in anxiety, negative mood, hallucinations and persecutory ideation right as relapses occur, participants also reported significant gradual increases, week-by-week, in persecutory ideation and hallucination in the lead up to relapses. Though brief and easy to administer, self-reported symptoms could provide critical information for remote measurement-based care management potentially aiding in detecting elevated risk and monitoring response to interventions (43). Applying this approach in psychiatry would mirror preventive interventions for physical health, for example, the treatment of risk factors for cardiovascular disease to prevent heart attacks rather than initiating treatment after they occur. If deployed proactively, psychiatric interventions could similarly reduce the significant impact of SSDs on healthcare systems and the lives of those affected.

## Data Availability Statement

The datasets presented in this study can be found in online repositories. The names of the repository/repositories and accession number(s) can be found below: The data analyzed for this study can be found at: https://www.mh4mh.org/eureka-data.

## Ethics Statement

The studies involving human participants were reviewed and approved by IRBs of Dartmouth College (#24356) and Northwell Health/Long Island Jewish Medical Center (#14-100B) and registered as a clinical trial (#NCT01952041). The patients/participants provided their written informed consent to participate in this study.

## Author Contributions

BB conducted the literature search, conducted data analysis, and wrote the first and subsequent revised drafts of the manuscript. KH helped devise the data analytic strategy and assisted in generation of figures. DB-Z, AC, TC, and JK oversaw the study resulting in this publication, including drafting the grant application, designing the study protocol, and supervising research staff managing study activities. AC and TC developed and refined mobile technologies employed. DB-Z supervised the drafting and revising of the manuscript. All authors contributed to and approved the final version of the manuscript.

## Conflict of Interest

DB-Z has an intervention content licensing agreement with Pear Therapeutics and has financial interest in FOCUS technology. He has consulted for Trusst Health Inc., eQuility, and Otsuka Pharmaceuticals Ltd. TC is a co- founder and holds equity stake at HealthRhythms Inc. JK has been a consultant for or received honoraria from Alkermes, Allergan, Dainippon Sumitomo, H. Lundbeck. Intracellular Therapies, Janssen Pharmaceutica, Johnson and Johnson, LB Pharmaceuticals, Merck, Minerva, Neurocrine, Otsuka, Reviva, Roche, Saladex, Sunovion, Takeda and Teva. He has received grant support from Otsuka, Lundbeck, and Janssen. JK is also a shareholder in Vanguard Research Group, LB Pharmaceuticals, Inc. and North Shore Therapuetics. KH has been a consultant for Pear Therapeutics. The remaining authors declare that the research was conducted in the absence of any commercial or financial relationships that could be construed as a potential conflict of interest.
